# Metagenomic Analysis of Antarctic Ocean near the King Sejong Station Reveals the Diversity of Carotenoid Biosynthetic Genes

**DOI:** 10.3390/microorganisms12020390

**Published:** 2024-02-15

**Authors:** Woo Yeon Cho, Pyung Cheon Lee

**Affiliations:** Department of Molecular Science and Technology, Ajou University, Woncheon-dong, Yeongtong-gu, Suwon 16499, Republic of Korea; wycho418@ajou.ac.kr

**Keywords:** metagenome, carotenoids, microbial diversity

## Abstract

Carotenoids, biotechnologically significant pigments, play crucial biological roles in marine microorganisms. While various environments have been explored to understand the diversity of carotenoids and their biosynthesis, the Antarctic Ocean remains relatively under-investigated. This study conducted a metagenomic analysis of seawater from two depths (16 and 25 m) near the King Sejong Station in the Antarctic Ocean. The analysis revealed a rich genetic diversity underlying C40 (astaxanthin, myxol, okenone, spheroidene, and spirilloxanthin), C30 (diaponeurosporene, diapolycopene, and staphyloxanthin), and C50 (C.p. 450) carotenoid biosynthesis in marine microorganisms, with notable differential gene abundances between depth locations. Exploring carotenoid pathway genes offers the potential for discovering diverse carotenoid structures of biotechnological value and better understanding their roles in individual microorganisms and broader ecosystems.

## 1. Introduction

Carotenoids encompass a diverse spectrum of organic pigments, ranging from yellows to brilliant oranges and deep reds [1,2]. These compounds offer more than just coloration; they act as precursors to several hormones and play multiple biological roles, including light-harvesting, photoprotection, and providing distinct hues to various organisms [3,4,5,6,7,8,9,10]. From a biotechnological perspective, specific carotenoids such as β-carotene, lycopene, and lutein have predominantly served as food colorants, antioxidants, and essential components in animal feed [11,12,13]. In addition to their conventional applications, these pigments have found practical uses in the fields of nutraceuticals, cosmetics, and pharmaceuticals [8,14,15]. The marine environment, abundant with diverse microorganisms, holds significant potential for the exploration of novel carotenoid-producing organisms. 

In the Antarctic Ocean, carotenoids are ecologically pivotal, functioning as essential bioindicators of environmental transformations. The extreme conditions prevailing in the Antarctic, characterized by severe cold, intense ultraviolet radiation, and extensive sea ice, pose distinctive challenges for carotenoid research. A comprehensive analysis of the spatial distribution, abundance, and heterogeneity of carotenoids would elucidate the complex adaptive mechanisms of Antarctic marine microbes. Such understanding would not only contribute to ecological knowledge but also catalyze the advancement of novel biotechnological applications of these compounds, underscoring their potential in bioprospecting endeavors [16,17,18,19].

While numerous carotenoid structures, especially those derived from microbial sources, have been identified, significant gaps still exist in our understanding of the genetic and enzymatic processes governing their biosynthesis [20]. The rapid expansion of metagenomics, supported by an increasing repository of microbial genomic sequences, provides an opportunity to unravel the enigmatic biosynthetic pathways of natural carotenoids [21]. This genomic wealth has the potential to facilitate the metabolic engineering of distinctive and economically beneficial carotenoid derivatives. Furthermore, exploring the involvement of marine microorganisms in carotenoid biosynthesis presents a promising avenue for investigation. However, the exploitation of this vast structural diversity is currently limited by the deficiency of specific and efficacious enzymatic catalysts. These organisms are likely to possess many yet-to-be-discovered genes essential for carotenoid production [22], which may lead to the creation of new carotenoid variants and expand our understanding of their wide-ranging biological functions.

In consideration of these factors, our research begins with a metagenomic analysis at two distinct depths near King Sejong Station in the Antarctic Ocean. This study aims to decode the genetic intricacies underlying carotenoid biosynthesis in Antarctic Ocean microorganisms, thereby enriching the scientific conversation within this domain.

## 2. Materials and Methods

### 2.1. Sample Information

Seawater samples were collected from the Antarctic Ocean near King Sejong Station. Approximately 1 L of seawater was collected at depths of 16 and 25 m. The samples were immediately filtered through 0.22 µm Sterivex filters to capture microbial biomass. Filters were flash-frozen in liquid nitrogen and stored at −80 °C until further processing.

### 2.2. DNA Extraction and Illumina Shotgun Sequencing

Microbial DNA was extracted from the filters using the DNeasy PowerWater Sterivex Kit (Qiagen, Hilden, Germany) following the manufacturer’s guidelines. DNA concentration and purity were assessed using a spectrophotometer (Eon microplate spectrophotometer, BioTek, Shoreline, WA, USA). Genomic DNA libraries were prepared using the Nextera XT DNA Library Preparation Kit (Illumina, San Diego, CA, USA). Libraries were quantified using the Qubit dsDNA HS Assay Kit and sequenced on an Illumina NovaSeq 6000 platform to generate 150 bp paired-end reads.

### 2.3. Quality Control and Data Preprocessing

Raw sequencing reads were subjected to quality control using FastQC (version 0.11.2). Low-quality reads and adapter sequences were trimmed using Trimmomatics v0.39 [23]. The statistics for the number of reads and bases are summarized in Table 1.

### 2.4. Metagenomic Assembly and Annotation with SqueezeMeta

The quality-filtered reads were processed using SqueezeMeta-v1.6 [24] for co-assembly, gene prediction, and functional annotation. The SqueezeMeta pipeline was run in the “coassembly” mode with MEGAHIT, which performs co-assembly of reads from all samples to generate a single set of contigs. Prodigal was used for gene prediction, and the genes were annotated using the built-in databases in SqueezeMeta, which include KEGG, COG, and Pfam. The pipeline uses Kraken 2 for taxonomic classification of the contigs and DIAMOND for functional annotation against the KEGG database. The internal tools of SqueezeMeta were used to perform differential abundance analysis of genes, taxa, and functions across different samples.

### 2.5. Statistical Analysis

Differential abundance analysis of carotenoid biosynthetic genes was performed within the SqueezeMeta pipeline using built-in statistical tools.

### 2.6. Data Availability

All raw sequencing data, along with the assembled and annotated metagenomes, have been deposited in the NCBI Sequence Read Archive (SRA) under accession number SRR26901671.

## 3. Results

### 3.1. Metagenomic Insights into Microbial Diversity of the Antarctic Ocean

**Figure 1:**
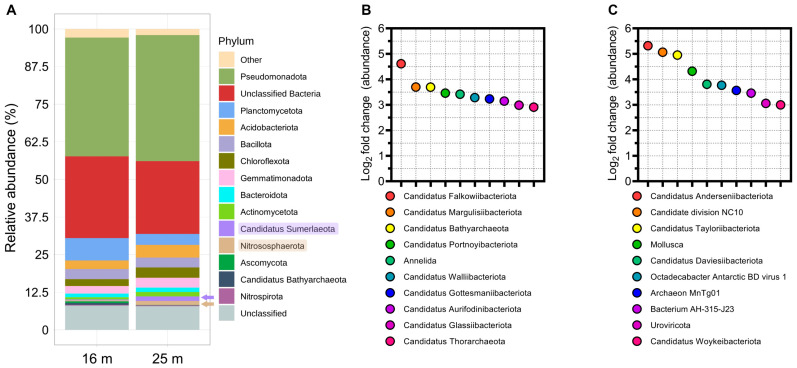
Relative microbial abundances at depths of 16 and 25 m. (**A**) Overall relative distribution of microbial taxa by phylum. (**B**) Phyla with differentially higher dominance at 16 m relative to 25 m, quantified using transcripts per million (TPM). (**C**) Phyla with differentially higher dominance at 25 m relative to 16 m, quantified using TPM.

Metagenomic analysis was employed to characterize the marine microbial diversity at two distinct vertical depths (16 and 25 m) at the phylum level. The predominance of *Pseudomonadota* was observed at both depths, followed by an assemblage of unclassified bacteria, namely *Planctomycetota*, *Acidobacteriota*, and *Bacillota* (Figure 1A), corroborating findings from previous studies [25]. Intriguingly, the phylum *Candidatus Sumerlaeota*, which has only recently been described and seldom reported [26], along with *Nitrososphaerota* [27], demonstrated a higher relative abundance at the 25 m depth compared to 16 m. Additionally, differential microbial abundance analysis, employing transcripts per million (TPM) as a metric, identified unique *Candidatus* bacterial and archaeal taxa at both depths (Figure 1B,C).

### 3.2. Diversity of Carotenoid Biosynthetic Enzymes in the Antarctic Ocean Revealed by Metagenomic Analysis

**Figure 2:**
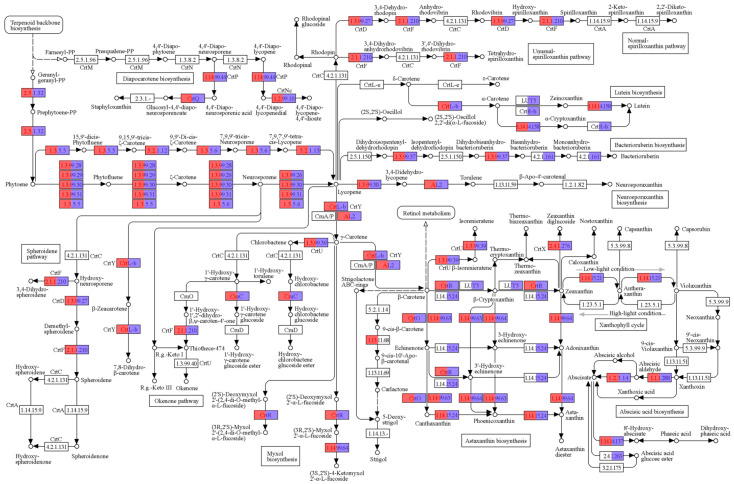
Carotenoid biosynthesis pathway genes identified from metagenomic data annotated with KEGG ID (ko00906). Carotenogenic genes found at 16 m are highlighted in red and those at 25 m in purple. The pathway diagram was sourced from the KEGG website.

Metagenomic analysis revealed a diverse array of genes encoding enzymes associated with the carotenoid biosynthesis pathway at depths of 16 and 25 m, annotated according to the KEGG pathway database (ID ko00906) and illustrated in Figure 2. These carotenogenic genes were broadly present across both sampled depths, with certain genes exclusively identified at specific depths (in Figure 2, genes unique to 16 m are denoted in red and genes unique to 25 m in purple).

**Figure 3:**
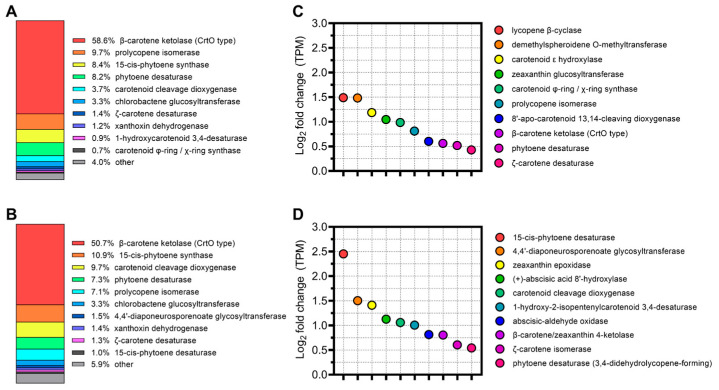
Distribution of carotenoid biosynthesis pathway genes at depths of 16 and 25 m. (**A**) Profile of gene distribution related to the carotenoid biosynthesis pathway at 16 m. (**B**) Profile of carotenogenic gene distribution at 25 m. (**C**) Genes showing differentially higher abundance at 16 m as determined by TPM. (**D**) Genes with differentially higher abundance at 25 m, also quantified using TPM.

Subsequently, the relative abundance of these genes was quantified. Notably, genes encoding β-carotene ketolase, which is essential for the biosynthesis of C40 carotenoids, was predominant at both depths, representing 58.6% of the gene population at 16 m (Figure 3A) and 50.7% at 25 m (Figure 3B). Other genes in the pathway exhibited a varied presence, accounting for between 10.9% and 0.40% of the carotenogenic gene population. Further differential abundance analysis using TPM identified several significantly more abundant carotenogenic genes at the 16 m depth. These include genes encoding lycopene β-cyclase and zeaxanthin glucosyltransferase, showing more than a two-fold increase compared to the 25 m depth (Figure 3C). In contrast, genes encoding enzymes such as 15-cis-phytoene desaturase and carotenoid cleavage dioxygenase were found to be more than twice as abundant at 25 m (Figure 3D).

### 3.3. C40 Carotenoid Biosynthetic Pathways in the Antarctic Ocean as Elucidated by Metagenomic Analysis

#### 3.3.1. Assessment of the Prevalence of C40 Astaxanthin Biosynthesis Genes in Antarctic Microbial Communities

**Figure 4:**
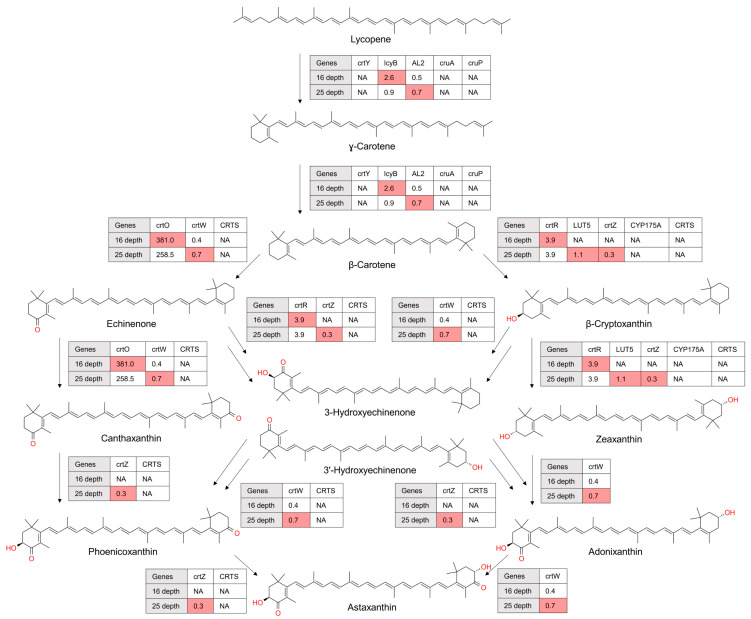
Astaxanthin biosynthesis from lycopene as revealed in metagenomic data. Each enzymatic step was annotated with its corresponding KEGG (Kyoto Encyclopedia of Genes and Genomes) identifier. Color coding was employed to signify enzymes with a higher gene abundance at specific depths. The absence of genes encoding enzymes was denoted by ‘NA’, indicating a lack of detectable genes at the respective depths. The numbers enclosed in boxes reflect carotenogenic gene abundance quantified by TPM (transcripts per million), offering a quantitative measure of gene abundance.

Astaxanthin, a keto-carotenoid of significant biotechnological interest, is synthesized via the C40 carotenoid biosynthetic pathway [28]. Microbial inhabitants of the Antarctic Ocean utilize unique enzymatic processes to produce astaxanthin, which confers survival advantages under extreme conditions, such as high UV radiation and low temperatures [29]. Our metagenomic analysis focused on the genes associated with the astaxanthin biosynthetic pathway, which extends from the formation of phytoene to the final product, astaxanthin (Figure 4). Notable genes encoding β-carotene hydroxylase (CrtZ) and β-carotene ketolase (CrtW), which catalyzes the final step in the astaxanthin biosynthesis, were more abundant or uniquely present at 25 m.

#### 3.3.2. Assessment of the Prevalence of C40 Myxol Biosynthesis Genes in the Microbiota of the Antarctic Ocean 

Myxol, a carotenoid derivative, undergoes synthesis via the C40 carotenoid biosynthetic pathway, involving distinct enzymatic steps that culminate in the formation of myxoxanthophyll—a glycosylated variant of myxol. This biosynthetic route is pivotal for microbial communities in the extreme environments of the Antarctic Ocean, aiding their adaptation and ecological prowess [30]. 

**Figure 5:**
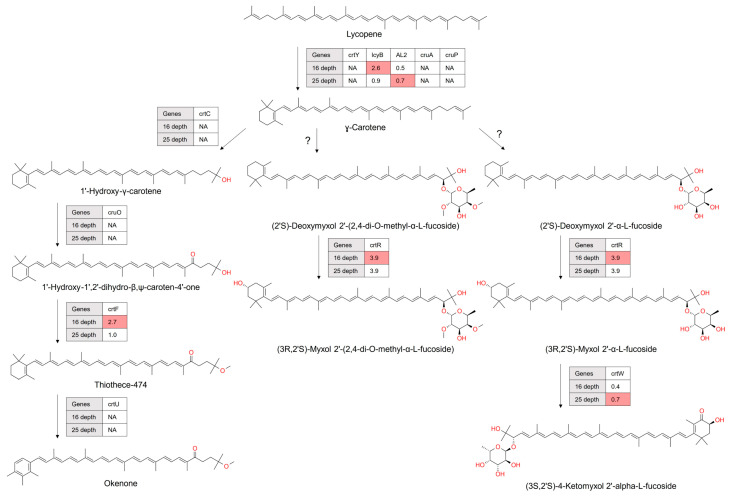
Biosynthesis of okenone and myxol as revealed by metagenomic analysis. A color-coded scheme highlights carotenogenic genes with increased abundance at specific depths. ‘NA’ denotes the absence of detectable genes for certain enzymes at the respective depths. Enclosed within boxes are numerical values representing the gene abundance of enzymes, quantified in transcripts per million (TPM), providing a quantitative assessment of gene abundance. Cases flagged with a question mark indicate missing genes encoding enzymes such as *O*-glycosyltransferase (CruG) in the carotenoid biosynthesis pathway (ko00906).

In our metagenomic study, we identified genes encoding key enzymes involved in converting typical carotenoid precursors into the structurally distinct myxol compounds (Figure 5). Carotenogenic genes such as genes encoding β-carotene hydroxylase (CrtR) and β-carotene/zeaxanthin 4-ketolase (CrtW or BKT) were prevalent at both depths. However, the gene encoding O-glycosyltransferase (CruG), crucial for sugar addition in myxol biosynthesis, was conspicuously absent at both depths.

#### 3.3.3. Assessment of the Prevalence of C40 Okenone Biosynthesis Genes in the Antarctic Ocean Microbiomes

Okenone, a ketocarotenoid with a unique pink-to-purple color, is synthesized through the C40 carotenoid biosynthetic pathway. This pathway plays a crucial role in the Antarctic Ocean’s microbiomes, representing a complex biochemical adaptation essential for survival in harsh photic environments [31]. The synthesis of okenone involves a sequence of enzymatic reactions starting from lycopene. These reactions include the specific introduction of hydroxy/keto groups and an χ-end group, which are pivotal in defining okenone’s chemical structure (Figure 5). Metagenomic analysis indicates the absence of genes encoding carotenoid 1,2-hydratase (CrtC), 1’-hydroxy-γ-carotene C-4’ ketolase (CrtO), and carotenoid χ-ring synthase (CrtU) at both sampled depths. Contrastingly, a gene encoding *O*-methyltransferase (CrtF) showed a higher abundance at 16 m compared to 25 m. 

#### 3.3.4. Assessment of the Prevalence of C40 Spheroidene Biosynthesis Genes in the Antarctic Ocean Microbiota

**Figure 6:**
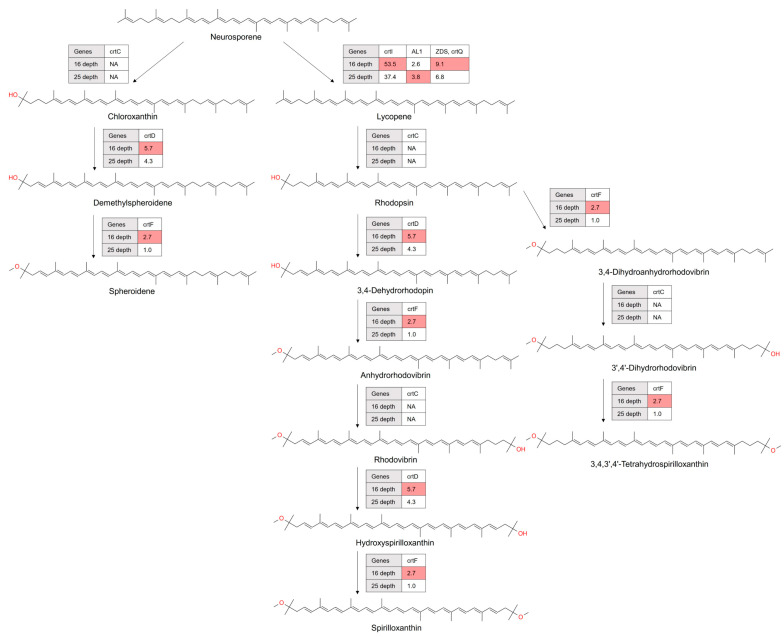
Spheroidene and spirilloxanthin (and 3,4,3′,4′-terahydrospirilloxanthin) biosynthesis as revealed by metagenomic data analysis. Each enzymatic step was documented with its corresponding KEGG identifier. Color coding shows carotenogenic genes with higher abundance at specific depths. The absence of genes encoding enzymes was indicated by ‘NA’, representing the lack of detectable genes at those depths. Numerical values enclosed within boxes denote the gene abundance of enzymes, measured in transcripts per million (TPM), offering a quantitative assessment of gene abundance.

Spheroidene is a carotenoid of the C40 family, known for its contribution to the photosynthetic apparatus of purple bacteria and its role as a photoprotective agent. Within the extreme photic conditions of the Antarctic Ocean, the biosynthesis of spheroidene suggests a highly specialized adaptation mechanism [32]. Our metagenomic analysis indicated that genes encoding C-3′,4′-desaturase (CrtD) and *O*-methyltransferase (CrtF) were more abundantly presented at the 16 m depth compared to 25 m (Figure 6), suggesting a stratification in biosynthetic activity that would correlate with environmental gradients, such as light or oxidative stress. Notably, gene encoding hydroxyneurosporene synthase (CrtC), which is critical in spheroidene biosynthesis, was not detected at both depths.

#### 3.3.5. Assessment of the Prevalence of C40 Spirilloxanthin Biosynthesis Genes in the Antarctic Ocean Microbiota

Spirilloxanthin, another member of the C40 carotenoid family, is recognized for its significance in bacterial photosynthetic processes and membrane stabilization [33]. The spirilloxanthin biosynthetic pathway is a series of enzymatic reactions that convert neurosporene into spirilloxanthin. This pathway is commonly found in purple non-sulfur bacteria like *Rhodospirillum rubrum*. Spirilloxanthin is synthesized through a cascade of enzymatic reactions starting from the precursor neurosporene: lycopene synthase (CrtI), carotenoid-1,2-hydratase (CrtC), 1-hydroxycarotenoid 3,4-desaturase (CrtD), and *O*-methyltransferase (CrtF). This cascade facilitates sequential modifications that transform lycopene into rhodopsin, 3,4-dehydrorhodopin or 3,4-dihydroanhydrorhodovibrin, and eventually spirilloxanthin and 3,4,3′,4′-terahydrospirilloxanthin (Figure 6).

### 3.4. C30 and C50 Carotenoid Biosynthetic Pathways of Antarctic Ocean Microbial Communities

#### 3.4.1. Assessment of the Prevalence of C30 Carotenoid Biosynthesis Genes in Antarctic marine Microorganisms

C30 carotenoids, while less prevalent than C40 variants, are intriguing due to their unique synthesis in specific microbial strains. These strains produce diaponeurosporene/diapolycopene and derivatives like staphyloxanthin [34,35]. The primary biosynthetic mechanism for these molecules involves the head-to-head condensation of two C15 farnesyl pyrophosphate (FPP) units, forming a 30-carbon skeleton. Alternatively, some pathways utilize the condensation of a C10 geranyl pyrophosphate (GPP) with a C20 geranylgeranyl pyrophosphate (GGPP) [36].

**Figure 7:**
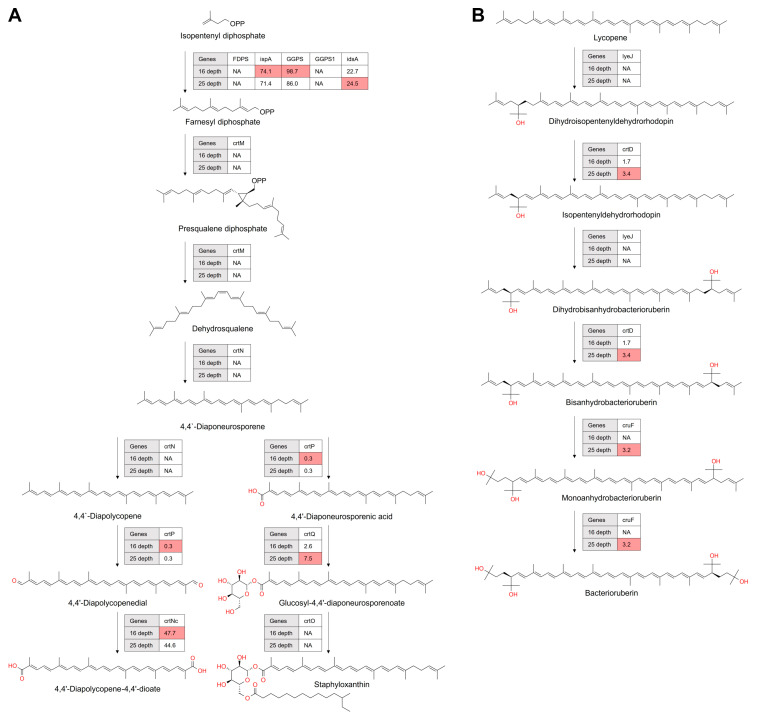
The biosynthetic pathways of C30 carotenoids (**A**) and C50 carotenoids (**B**) depicted based on metagenomic data. Each enzymatic step is annotated with its corresponding KEGG identifier. Color coding shows carotenogenic genes with elevated abundance at specific depths. The absence of genes encoding enzymes is marked by ‘NA’, denoting undetectable genes at those depths. Numerical values within boxes represent transcripts per million (TPM), offering a quantitative measure of gene abundance of carotenogenic enzymes.

Our metagenomic study revealed the absence of genes encoding key enzymes (CrtM and CrtN) in the diaponeurosporene/diapolycopene biosynthesis pathway (see Figure 7A). This indicates a scarce presence of C30 carotenoid-producing microorganisms in the Antarctic marine samples we analyzed [34]. However, genes encoding enzymes involved in modifying the C30 carotenoid backbone, such as 4,4-diaponeurosporene oxidase (CrtP), 4,4′-diapolycopenoate synthase (CrtNc), and 4,4′-diaponeurosporenoate glycosyltransferase (CrtQ), were detected at both depths. The only exception was a gene encoding glycosyl-4,4′-diaponeurosporenoate acyltransferase (CrtO), which was not found.

#### 3.4.2. Assessment of the Prevalence of C50 Carotenoid Biosynthesis Genes in Antarctic marine Ecosystems

C50 carotenoids, though less prevalent than their C40 counterparts, are of significant interest due to their presence in specific microbial strains. These strains are known for producing unique compounds such as decaprenoxanthin [37,38], bacterioruberin [39,40], and C.p. 450 [41]. Characteristic of C50 carotenoids is their extended 50-carbon skeletons, distinguishing them from the standard 40-carbon carotenoid framework. Our metagenomic study revealed varied distribution patterns of genes involved in bacterioruberin synthesis (Figure 7B). Genes encoding enzymes such as 1-hydroxy-2-isopentenylcarotenoid 3,4-desaturase (CrtD) and bisanhydrobacterioruberin hydratase (CrtF) showed higher abundances at a depth of 25 m. Notably, we did not detect the lycopene elongase/hydratase (LyeJ) gene in the deep-water samples.

## 4. Discussion

This study utilizes metagenomic analysis to decipher the genetic complexities of carotenoid biosynthesis in the Antarctic Ocean near King Sejong Station, aiming to enrich the understanding of carotenogenic microbial diversity and the array of biological functions they mediate. Metagenomic analysis reveals a diverse array of carotenogenic genes at 16 and 25 m in the Antarctic Ocean, with β-carotene ketolase predominating at both depths. Differential abundance analysis uncovers significant variations in the presence of specific carotenogenic genes between the two depths, indicating depth-dependent variations in carotenoid biosynthesis pathways. The profiling unveils a rich tapestry of gene abundance that sustains carotenoid production under the unique physicochemical conditions found in Antarctic waters, reflective of a highly adaptive microbial population. 

Astaxanthin, known for providing survival advantages under high UV radiation and low temperatures, exhibits depth-specific adaptations in its biosynthesis [28,29]. Genes encoding CrtZ and CrtW show increased abundance at 25 m, indicating environmental adaptation mechanisms of astaxanthin-producing microbes to the depth of the Antarctic Ocean. 

Spirilloxanthin, crucial in bacterial photosynthetic processes and membrane stabilization, exhibits depth-specific adaptations [33]. Genes encoding CrtD and CrtF show higher abundance at 16 m, suggesting a targeted response to varying light conditions and oxidative stress levels with increased depth. This niche-specific adaptation enhances survival in low-light conditions, where spirilloxanthin plays a pivotal role in photosynthetic efficiency. 

Bacterioruberin, known for protecting microorganisms against oxidative and temperature stress, shows varied distribution patterns of genes involved in its synthesis [39,40]. Genes encoding CrtD and CrtF exhibit higher abundance at 25 m depth, suggesting a depth-dependent adaptation to environmental stress. The absence of the gene encoding LyeJ in the deep-water samples aligns with existing research on the capabilities of halobacteria and related marine microorganisms in producing C50 carotenoids [42,43].

## 5. Conclusions

This investigation sheds light on the complex mechanisms underlying carotenoid biosynthesis within polar marine ecosystems, highlighting the variability encountered at various oceanic depths. Our research not only enhances the scientific comprehension of marine biochemistry but also reveals the potential biotechnological applications of these carotenogenic marine microorganisms and their biosynthesis genes, offering promising prospects for industrial innovation. Despite the challenges encountered in our metagenomic approach, especially regarding comprehensive sequence coverage and sample volume, we have successfully established a foundation for subsequent research into the diversity of carotenoid biosynthesis within cold marine ecosystems.

## Figures and Tables

**Table 1:** Sequencing statistics at different depths of coverage.

Metric	16 m Depth	25 m Depth
Number of reads	12,362,180	11,486,332
Number of bases	3,102,907,180	2,883,069,332

## Data Availability

Data are contained within the article.
